# Substantia nigra echogenicity is associated with serum ferritin, gender and iron-related genes in Parkinson’s disease

**DOI:** 10.1038/s41598-020-65537-5

**Published:** 2020-05-26

**Authors:** Kai Li, Yi-Lun Ge, Chen-Chen Gu, Jin-Ru Zhang, Hong Jin, Jiao Li, Xiao-Yu Cheng, Ya-Ping Yang, Fen Wang, Ying-Chun Zhang, Jing Chen, Cheng-Jie Mao, Chun-Feng Liu

**Affiliations:** 1 Department of Neurology, the Second Affiliated Hospital of Soochow University, Suzhou, Jiangsu China; 2 Institute of Neuroscience, Soochow University, Suzhou, Jiangsu China; 3 Department of Ultrasound, the Second Affiliated Hospital of Soochow University, Suzhou, Jiangsu China

**Keywords:** Parkinson's disease, Parkinson's disease

## Abstract

Substantia nigra (SN) hyperechogenicity is present in most Parkinson’s disease (PD) cases but is occasionally absent in some. To date, age, gender, disease severity, and other factors have been reported to be associated with SN hyperechogenicity in PD. Previous studies have discovered that excess iron deposition in the SN underlies its hyperechogenicity in PD, which may also indicate the involvement of genes associated with iron metabolism in hyperechogenicity. The objective of our study is to explore the potential associations between variants in iron metabolism-associated genes and SN echogenicity in Han Chinese PD. Demographic profiles, clinical data, SN echogenicity and genotypes were obtained from 221 Han Chinese PD individuals with a sufficient bone window. Serum ferritin levels were quantified in 92 of these individuals by immunochemical assay. We then compared factors between PD individuals with SN hyperechogenicity and those with SN hypoechogenicity to identify factors that predispose to SN hyperechogenicity. Of our 221 participants, 122 (55.2%) displayed SN hyperechogenicity, and 99 (44.8%) displayed SN hypoechogenicity. Gender and serum ferritin levels were found to be associated with SN hyperechogenicity. In total, 14 genes were included in the sequencing part. After data processing, 34 common single nucleotide polymorphisms were included in our further analyses. In our data, we also found a significantly higher frequency of *PANK2* rs3737084 (genotype: OR = 2.07, *P* = 0.013; allele: OR = 2.51, *P* = 0.002) in the SN hyperechogenic group and a higher frequency of *PLA2G6* rs731821 (genotype: OR = 0.45, *P* = 0.016; allele: OR = 0.44, *P* = 0.011) in the SN hypoechogenic group. However, neither of the two variants was found to be correlated with serum ferritin. This study demonstrated that genetic factors, serum ferritin level, and gender may explain the interindividual variability in SN echogenicity in PD. This is an explorative study, and further replication is warranted in larger samples and different populations.

## Introduction

Parkinson’s disease (PD) is the second most common neurodegenerative disease, characterized by motor and non-motor symptoms such as bradykinesia, rigidity, resting tremor, hyposmia, and rapid eye movement sleep behaviour disorder^1,2^. Substantia nigra hyperechogenicity (SN+) under transcranial sonography (TCS) is a common feature of PD that enables reliable diagnosis of PD with high sensitivity and specificity^3,4^. SN+ is found in approximately 60–90% of individuals with PD; however, it is absent in some cases^5–7^, indicating potential interindividual variability. To date, many factors have been found to be associated with SN+ in PD, including age, gender, disease severity, and other factors^8–10^.

At present, the detailed mechanism underlying SN+ is not very clear^11–13^. However, postmortem studies have revealed that the SN+ area is positively correlated with abnormal iron accumulation in PD^14,15^, indicating that dysfunction of iron metabolism may be involved. *PANK2*, *COASY*, *PLA2G6*, and other genes related to iron homeostasis have been reported to cause diseases related to abnormal iron accumulation, such as neurodegeneration with brain iron accumulation (NBIA) and infantile neuroaxonal dystrophy (INAD). In the present study, we aimed to compare the clinical characteristics and genotypes of PD individuals with different SN echogenicities to determine whether there are genetic factors that play a role in SN+ in PD. This is an explorative study and requires further replication.

## Methods

### Study participants

We recruited 250 consecutive patients with PD (aged from 40 to 83 years) from the Department of Neurology of the Second Affiliated Hospital of Soochow University (Suzhou, China) from November 2011 to September 2018. Twenty-nine of the 250 individuals were excluded from the study because of an insufficient bone window, and 221 individuals were finally included in the study. All of the study participants fulfilled either the 2015 Movement Disorder Society clinical diagnostic criteria^16^ or the UK Brain Bank criteria for PD^17^. The investigation protocol was approved by the ethics committee of the Second Affiliated Hospital of Soochow University. Detailed clinical data including demographics, the Unified Parkinson Disease Rating Scale (UPDRS) Part III in the “on” state, the modified Hoehn and Yahr stage in the “off” state^18^ and peripheral blood samples were collected after written informed consent was obtained from all the participants and their legally authorized representatives. All methods were performed in accordance with the relevant guidelines and regulations.

### Transcranial sonography

TCS examinations were performed according to our previous study^19^ within 1 week after clinical assessment. The brain was insonated through the temporal acoustic bone window in the orbitomeatal line using a transducer (Acuson Sequoia 512, Siemens, Germany) with an emitting frequency of 2.5 MHz. The penetration depth was adjusted to 14 to 16 cm, and the dynamic range was 45 dB to 55 dB^3,19,20^. The hyperechogenic signal at the anatomical site of the SN within the hypoechogenic mesencephalic brainstem was visualized to its greatest extent, and the image was frozen and magnified by 3- to 4-fold for more precise measurement^3,20^. The area of the SN was manually encircled with the cursor, and the planimetric area was calculated automatically^20^. SN+ was defined by a hyperechogenic area within the left or right SN equal to or greater than 0.20 cm^2^, whereas SN hypoechogenicity (SN−) was defined as both left and right SN hyperechogenic areas less than 0.20 cm^2^ ^3^. The TCS was performed in a darkened room by the same experienced clinician who was blinded to the individuals’ clinical status to eliminate any bias in the results of the examination^3^.

### High-throughput sequencing

Genomic DNA was extracted from the blood samples using the QIA ampDNA Blood Maxi Kit (QIAGEN, Valencia, CA, USA). Two genes in the iron metabolic pathway (*TF* and *SLC11A2*)^21,22^ and 12 genes associated with NBIA^23–26^ (*FTL*, *CP*, *PANK2*, *COASY*, *PLA2G6*, *C19orf12*, *FA2H*, *ATP13A2*, *WDR45*, *DCAF17*/*C2orf37*, *SCP2*, and *GTPBP2*) were included in our genotype analysis. One hundred seventeen of the samples were sequenced by target-region sequencing, and the other 104 samples were sequenced by whole-exome sequencing, as described in our previous study^27^. Single nucleotide polymorphisms (SNPs) and insertions and deletions (Indels) located within the exons and exon-intron boundaries of the gene were then identified as described previously^27^.

After variant calling and annotation, the data were compared to the 1000 Genome Project East Asian database (GRCh37) and to our in-house data to distinguish common and rare variants. Linkage disequilibrium was estimated using SHEsis (online version)^28,29^, and SNPs with r^2^ ≥ 0.80 were defined as being in linkage disequilibrium.

### Serum ferritin assessment

Fasting blood samples for serum ferritin quantification were obtained from 101 of the 221 participants after an overnight fast of at least 12 h. Ferritin levels in the serum samples were quantified by immunochemical assay (Cobas 6000–1, Roche, Roche Diagnostics International Ltd, Switzerland) in the radio-immuno imaging centre of the Second Affiliated Hospital of Soochow University within 1 h of sample collection. Nine participants with acute or chronic inflammation or tumours were excluded^30^ from further association analyses of SN echogenicity, genotype and ferritin.

### Statistical analyses

For univariate analyses, variables were tested for normality by the Kolmogorov-Smirnov test or Shapiro-Wilk’s test. Differences in metric variables between the SN+ group and SN− group were evaluated by Student’s *t*-test or Mann-Whitney U test. Dichotomous variables were compared between the two groups by the chi-square test or Fisher’s exact test. We assessed the associations among demographic profiles, ferritin levels, and common SNPs by binary logistic regression of SN echogenicity.

All statistical tests were two sided with *P* < 0.05 as the threshold for statistical significance. All tests were performed using IBM SPSS Statistics, version 25.0, 64-bit (IBM Corporation, Armonk, NY, USA).

## Results

### Demographics, clinical features, TCS assessment, and genetic findings

All 221 participants underwent TCS examination and high-throughput DNA sequencing. The study cohort included 138 (62.4%) men and 83 (37.6%) women. Among 221 participants, 122 (55.2%) were SN+ (unilateral SN area on either side ≥ 0.20 cm^2^), and the other 99 were SN− (SN area on both sides <0.20 cm^2^). The proportion of males was significantly higher among the individuals with SN+ than among those with SN− (*P* < 0.001), whereas age, disease duration, Hoehn and Yahr stage, and UPDRS-III ratings were similar between the two groups (Supplemental Table 1). Variants with minor allele frequency (MAF) < 0.15 were excluded from further analyses to increase our statistical power. We also excluded SNPs that were present because of linkage disequilibrium with another SNP within the same region. Then, 34 common SNPs were included (MAF ≥ 0.15), and 0 common Indel was included (MAF ≥ 0.15) in our further analysis.

**Table 1:** Genotype and allele analysis of 34 SNPs among 221 individuals with PD.

	Total (n = 221)	SN+ (n = 122)	SN− (n = 99)	OR	95% CI	*P*
Gender (Male, %)	138 (62.4%)	92 (75.4%)	46 (46.5%)	0.32	0.16–0.63	0.001^*^
Age (years)	62.5 ± 8.6	63.4 ± 8.2	61.4 ± 9.0	1.02	0.98–1.06	0.260^*^
PD Duration (years)	4.3 ± 3.6	4.3 ± 3.8	4.2 ± 3.2	1.01	0.91–1.13	0.833^*^
H-Y	2.1 ± 0.7	2.1 ± 0.7	2.1 ± 0.8	0.78	0.42–1.46	0.432^*^
UPDRS-III	25.0 ± 11.9	25.6 ± 12.7	24.4 ± 10.9	1.01	0.97–1.05	0.552^*^
rs731821 (CC/CA/AA)	122/87/12	75/44/3	47/43/9	0.45	0.24–0.86	0.016^*^
rs3737084 (CC/CG/GG)	132/74/15	62/49/11	70/25/4	2.07	1.17–3.67	0.013^*^
Gender (Male, %)	276 (62.4%)	184 (75.4%)	92 (46.5%)	0.31	0.20–0.49	<0.001^†^
Age (years)	62.5 ± 8.6	63.4 ± 8.2	61.4 ± 9.0	1.02	0.99–1.05	0.139^†^
PD Duration (years)	4.3 ± 3.5	4.3 ± 3.8	4.2 ± 3.2	1.01	0.94–1.09	0.791^†^
H-Y	2.1 ± 0.7	2.1 ± 0.7	2.1 ± 0.7	0.79	0.53–1.19	0.264^†^
UPDRS-III	25.0 ± 11.9	25.6 ± 12.7	24.4 ± 10.9	1.01	0.99–1.04	0.293^†^
rs731821 (C/A)	331/111	194/50	137/61	0.44	0.24–0.83	0.011^†^
rs3737084 (C/G)	338/104	173/71	165/33	2.51	1.39–4.53	0.002^†^

PD: Parkinson’s disease; SN+: Substantia nigra hyperechogenicity; SN−: Substantia nigra hypoechogenicity; OR: odds ratio; H-Y: Hoehn and Yahr stage (“off” state); UPDRS-III: Unified Parkinson Disease Rating Scale Part III (“on” state).

^*^P-values estimated from binary logistic regression models adjusted for age, gender, disease severity, and disease duration. The results of the other 32 SNPs are available in Supplemental Table 2.

^†^*P*-values estimated from binary logistic regression models adjusted for age, gender, disease severity, and disease duration. The results of the other 32 SNPs are available in Supplemental Table 3.

### Predisposing common SNPs among patients with SN+ and SN−

To determine if any of the 34 common SNPs were associated with the SN echogenicity status, we conducted a logistic regression analysis between the SN+ group and SN− group, considering age, gender, disease duration, and disease severity. We found a significantly higher frequency of *PANK2* rs3737084 (genotype: OR = 2.07, *P* = 0.013; allele: OR = 2.51, *P* = 0.002) in the SN+ group and a higher frequency of *PLA2G6* rs731821 (genotype: OR = 0.45, *P* = 0.016; allele: OR = 0.44, *P* = 0.011) in the SN− group (Table 1, Supplemental Tables 2 and 3). In addition, we also found that gender was associated with SN+ (genotype: OR = 0.32, *P* = 0.001; allele: OR = 0.31, *P* < 0.001) (Table 1, Supplemental Tables 2 and 3), indicating female gender as a protective factor, which is in accordance with the results of a previous study^8^.

**Table 2:** Genotype and allele analysis of rs731821 and rs3737084 among 92 PD individuals with serum ferritin data.

	Total (n = 92)	SN+ (n = 49)	SN− (n = 43)	OR	95% CI	*P*
Gender (Male, %)	57 (62.0%)	37 (75.5%)	20 (46.5%)	0.26	0.09–0.77	0.015^*^
Age (years)	63.7 ± 8.7	64.0 ± 7.9	63.3 ± 9.6	0.98	0.92–1.04	0.549^*^
PD Duration (years)	5.0 ± 3.8	5.1 ± 4.2	5.0 ± 3.3	1.11	0.94–1.31	0.233^*^
H-Y	2.3 ± 0.7	2.3 ± 0.8	2.3 ± 0.7	0.97	0.40–2.35	0.943^*^
UPDRS-III	28.1 ± 12.6	29.0 ± 14.3	27.1 ± 10.6	0.99	0.94–1.04	0.726^*^
Ferritin (μg/L)	186.9 ± 128.1	223.8 ± 150.8	144.9 ± 78.6	1.01	1.00–1.01	0.014^*^
rs731821 (CC/CA/AA)	51/36/5	33/16/0	18/20/5	0.18	0.07–0.50	<0.001^*^
rs3737084 (CC/CG/GG)	55/30/7	24/21/4	1931–09–03	2.84	1.13–7.13	0.026^*^
Gender (Male, %)	114 (62.0%)	74 (75.5%)	40 (46.5%)	0.29	0.14–0.60	<0.001^†^
Age (years)	63.7 ± 8.7	64.0 ± 7.8	63.3 ± 9.5	0.99	0.95–1.03	0.599^†^
PD Duration (years)	5.0 ± 3.8	5.1 ± 4.2	5.0 ± 3.3	1.06	0.95–1.18	0.306^†^
H-Y	2.3 ± 0.7	2.3 ± 0.8	2.3 ± 0.7	1.00	0.55–1.81	0.989^†^
UPDRS-III	28.1 ± 12.6	29.0 ± 14.2	27.1 ± 10.5	1.00	0.97–1.04	0.888^†^
Ferritin (μg/L)	186.9 ± 127.7	223.8 ± 150.0	144.9 ± 78.1	1.01	1.00–1.01	<0.001^†^
rs731821 (C/A)	138/46	82/16	56/30	0.20	0.08–0.48	<0.001^†^
rs3737084 (C/G)	140/44	69/29	71/15	3.45	1.41–8.45	0.007^†^

PD: Parkinson’s disease; SN+: Substantia nigra hyperechogenicity; SN−: Substantia nigra hypoechogenicity; OR: odds ratio; H-Y: Hoehn and Yahr stage (“off” state); UPDRS-III: Unified Parkinson Disease Rating Scale Part III (“on” state).

^*^P-values estimated from binary logistic regression models adjusted for age, gender, disease severity, and disease duration.

^†^*P*-values estimated from binary logistic regression models adjusted for age, gender, disease severity, and disease duration.

**Table 3:** Rs3737084, rs731821, and serum ferritin associations among 92 individuals with serum ferritin data.

	Gender (Male, %)	Age (years)	PD Duration (years)	H-Y	UPDRS-III	Ferritin (μg/L)
rs731821						
Total (n = 184)	114 (62.0%)	63.7 ± 8.7	5.0 ± 3.8	2.3 ± 0.7	28.1 ± 12.6	186.9 ± 127.7
‘C’ (n = 138)	84 (60.9%)	63.9 ± 8.7	4.9 ± 4.0	2.3 ± 0.8	29.3 ± 13.2	191.8 ± 131.0
‘A’ (n = 46)	30 (65.2%)	63.2 ± 8.6	5.5 ± 3.3	2.2 ± 0.6	24.8 ± 10.0	172.4 ± 117.4
OR	0.83	0.99	1.12	0.92	0.96	1.00
95% CI	0.39–1.76	0.95–1.03	1.01–1.24	0.49–1.70	0.92–1.00	0.39–1.76
*P*^†^	0.626	0.703	0.039	0.782	0.031	0.557
rs3737084						
Total (n = 184)	114 (62.0%)	63.7 ± 8.7	5.0 ± 3.8	2.3 ± 0.7	28.1 ± 12.6	186.9 ± 127.7
‘C’ (n = 140)	82 (58.6%)	63.4 ± 8.5	5.0 ± 3.8	2.3 ± 0.7	28.2 ± 13.2	188.4 ± 130.9
‘G’ (n = 44)	32 (72.7%)	64.8 ± 9.1	5.2 ± 3.9	2.3 ± 0.7	28.0 ± 10.9	182.2 ± 118.5
OR	0.52	1.01	1.02	0.97	1.00	1.00
95% CI	0.24–1.14	0.97–1.06	0.92–1.13	0.52–1.82	0.96–1.04	1.00–1.00
*P*^†^	0.102	0.527	0.734	0.931	0.991	0.557

PD: Parkinson’s disease; H-Y: Hoehn and Yahr stage; UPDRS-III: Unified Parkinson Disease Rating Scale Part III, OR: odds ratio; PD: Parkinson’s disease; CI: confidential interval.

^†^*P*-values estimated from binary logistic regression models adjusted for age, gender, disease severity and disease duration.

### Comparison of serum ferritin, rs3737084, and rs731821 between SN+ and SN−

Because serum ferritin has been shown to be associated with SN echogenicity^10^, we further included serum ferritin data in our logistic analysis of SN echogenicity referring to our candidate SNPs, i.e., rs3737084 and rs731821.

Inflammation and tumours are ever-known factors leading to ferritin increase, so we excluded patients with chronic inflammation or tumours from further analysis. In total, 101 of 221 PD had serum ferritin quantification, and 9 were excluded because of the comorbidity of inflammation or tumours. Among the 92 participants included in the serum ferritin analysis, 49 (53.3%) were SN+, and 43 (46.7%) were SN− (Supplemental Table 4).

In our data, considering age, gender, disease duration, disease severity and serum ferritin, we also found a significantly higher frequency of *PANK2* rs3737084 (genotype: OR = 2.84, *P* = 0.026; allele: OR = 3.45, *P* = 0.007) in the SN+ group and a higher frequency of *PLA2G6* rs731821 (genotype: OR = 0.18, *P* < 0.001; allele: OR = 0.20, *P* < 0.001) in the SN− group (Table 2). In addition, the individuals with SN+ had higher serum ferritin levels than those with SN− (*P* < 0.05; Table 2). We also found that gender was associated with SN+ (genotype: OR = 0.26, *P* = 0.015; allele: OR = 0.29, *P* < 0.001) (Supplemental Table 4).

### Analyses of the association of serum ferritin with rs3737084 and rs731821

To determine whether rs3737084 and rs731821 were associated with serum ferritin levels, we conducted a logistic regression of the alleles, considering gender, age, disease duration, and disease severity. We did not find that any of the alleles of the two variants were correlated with serum ferritin levels (*P* = 0.557 for each SNP; Table 3).

## Discussion

In 1995, Becker described structural changes in the SN of patients with PD for the first time^31^. Since then, TCS has become established as an important component of PD diagnosis^32^. TCS is used extensively in clinical practice, although it is not as accurate as expected^5–7^. SN echogenicity is heterogeneous among individuals with PD, appearing in approximately 60–90% of individuals^5–7^. To date, many factors have been linked to the variation in SN echogenicity, including age, gender, disease severity, serum ferritin, and other clinical factors^8–10^. Genetic factors have rarely been explored in relation to SN echogenicity^33–35^. We found that male gender and higher serum ferritin levels were associated with SN+, which is in accordance with previous findings by Zhou *et al*. and Yu *et al*.^8,10^. Those studies, like our own, mainly included Han Chinese individuals. Conducting similar studies considering other populations in the future is suggested.

We found that regardless of serum ferritin levels, there was a higher frequency of *PLA2G6* rs731821 in the SN− group and a higher frequency of *PANK2* rs3737084 in the SN+ group, indicating that genetic factors may play an important role in SN echogenicity. *PLA2G6* encodes a phospholipase (phospholipase A2, group IV) that localizes in the mitochondria and is important in membrane phospholipid remodelling, signal transduction, cell proliferation, and apoptosis^36–39^. Disruption of normal *PLA2G6* function could also lead to iron deposits^40,41^. In our study, we found that rs731821 was associated with SN−. This SNP locus is located within intron 2 of *PLA2G6*, which may affect its transcription or splicing, causing its dysfunction involving iron metabolism. *PANK2*, another NBIA causative gene, encodes an essential mitochondrial regulatory enzyme (pantothenate kinase 2) in a committed step of coenzyme A biosynthesis. Disruption of normal *PANK2* function could lead to iron accumulation in the brain by altering brain iron transport, mediated by alteration of ferroportin expression^42^, which is involved in PD pathology^43^. In our study, we found that *PANK2* rs3737084 is associated with SN+. This SNP locus is also located within exon 1 of *PANK2*, which is a missense variant leading to the 126^th^ amino acid residue alteration from glycine to alanine, which may cause the dysfunction of *PANK2* and be involved in iron metabolism. However, the detailed underlying mechanisms of the two SNPs remain unknown, and additional functional analyses are warranted in the future for further explanation.

Ferritin is important in the maintenance of iron homeostasis, mainly for iron storage and recycling^44^. SN+ PD were previously found to have elevated ferritin levels both in the SN hyperechogenic area and in the serum^10,45^. In our study, SN+ PD had higher serum ferritin levels than SN− PD, which is in accordance with previous studies^10,45^. However, we did not find an association between serum ferritin levels and *PLA2G6* rs731821 or *PANK2* rs3737084, suggesting that these two SNPs might influence SN echogenicity by a mechanism that may not involve serum ferritin.

This is an explorative study, and further validation of our findings is needed. To the best of our knowledge, this is the first study to provide evidence of the relationship between SN echogenicity and genetic factors in the Han Chinese population. Our study had some limitations, however. First, because of our limited sample size, we did not pay attention to rare and less common variants in our analyses, and we only had serum ferritin data for 92 of the participants, which limits our statistical power. Future studies with larger sample sizes will be able to include more rare genetic variants and have greater statistical power to detect relationships (such as the other genes associated with iron metabolism in our study) that our analysis might have missed. Second, we did not correct the *P*-values to a stricter standard, such as by Bonferroni or false discovery rate corrections, which could decrease the false positive rate with multiple comparisons. However, the two candidate SNPs identified in our analysis are located in *PLA2G6* and *PANK2*, which are both known to play important roles in iron metabolism-related genes, which may offer more evidence for our results. Third, the individuals in the SN+ group were more frequently male; however, given our limited sample size, we did not conduct subgroup analyses for men and women.

## Conclusion

Our study demonstrated that genetic factors, in addition to serum ferritin level and gender, might be associated with SN echogenicity in PD, which might explain the partial variability in SN echogenicity among different individuals. This is an explorative study, and further replication is warranted in larger samples and different populations.

## Supplementary information

Supplementary Table 1.

Supplementary Table 2.

Supplementary Table 3.

Supplementary Table4.
