# Supplementary material for: Substantia nigra echogenicity is associated with serum ferritin, gender and iron-related genes in Parkinson’s disease
Source: Sci Rep. 2020 May 26;10:8660. doi: 10.1038/s41598-020-65537-5 (PMC7250839; doi:10.1038/s41598-020-65537-5)
Supplement: Supplementary file 1 — Supplementary Table 1. [file 41598_2020_65537_MOESM1_ESM.docx]

Substantia nigra echogenicity is associated with serum ferritin, gender and iron-related genes in Parkinson's disease

Kai Li, MD, PhD,^1†^ Yi-Lun Ge, MD,^1†^ Chen-Chen Gu, MD,^1^ Jin-Ru Zhang, MD, PhD,^1^ Hong Jin, MD,^1^ Jiao Li, MD, PhD,^1^ Xiao-Yu Cheng, MD, PhD,^1^ Ya-Ping Yang, MD, PhD,^1^ Fen Wang, MD,^2^ Ying-Chun Zhang, MD, PhD,^3^ Jing Chen, MD, PhD,^1^ Cheng-Jie Mao, MD, PhD^1^ and Chun-Feng Liu, MD, PhD,^1,2*^

^1^Department of Neurology, the Second Affiliated Hospital of Soochow University, Suzhou, Jiangsu, China

^2^Institute of Neuroscience, Soochow University, Suzhou, Jiangsu, China

^3^Department of Ultrasound, the Second Affiliated Hospital of Soochow University, Suzhou, Jiangsu, China

^†^Kai Li and Yi-Lun Ge contributed equally to this work.

***Correspondence Author:**

Dr. Chun-Feng Liu, MD, PhD

Department of Neurology, the Second Affiliated Hospital of Soochow University, No.1055 Sanxiang Road, Suzhou, Jiangsu, 215004, P. R. China

Tel.: (86)512-6778-3307

E-mail: liuchunfeng@suda.edu.cn

**Supplemental Table 1.** Demographic and clinical features of the 221 individuals with PD.

|  | Total (n=221) | SN+ (n=122) | SN- (n=99) | *P* |
| --- | --- | --- | --- | --- |
| Gender (Male, %) | 138 (62.4%) | 92 (75.4%) | 46 (46.5%) | <0.001^a^ |
| Age (years) | 62.5 ± 8.6 | 63.4 ± 8.2 | 61.4 ± 9.0 | 0.103^b^ |
| PD Duration (years) | 4.3 ± 3.6 | 4.3 ± 3.8 | 4.2 ± 3.2 | 0.921^b^ |
| H-Y | 2.1 ± 0.7 | 2.1 ± 0.7 | 2.1 ± 0.8 | 0.772^b^ |
| UPDRS-III | 25.0 ± 11.9 | 25.6 ± 12.7 | 24.4 ± 10.9 | 0.701^b^ |

PD: Parkinson’s disease; SN+: Substantia nigra hyperechogenicity; SN-: Substantia nigra hypoechogenicity; H-Y: Hoehn and Yahr stage ("off" state); UPDRS-III: Unified Parkinson Disease Rating Scale Part III ("on" state).

^a^chi-square test.

^b^Mann-Whitney test.
