# Supplementary material for: Substantia nigra echogenicity is associated with serum ferritin, gender and iron-related genes in Parkinson’s disease
Source: Sci Rep. 2020 May 26;10:8660. doi: 10.1038/s41598-020-65537-5 (PMC7250839; doi:10.1038/s41598-020-65537-5)
Supplement: Supplementary file 2 — Supplementary Table 2. [file 41598_2020_65537_MOESM2_ESM.docx]

Substantia nigra echogenicity is associated with serum ferritin, gender and iron-related genes in Parkinson's disease

Kai Li, MD, PhD,^1†^ Yi-Lun Ge, MD,^1†^ Chen-Chen Gu, MD,^1^ Jin-Ru Zhang, MD, PhD,^1^ Hong Jin, MD,^1^ Jiao Li, MD, PhD,^1^ Xiao-Yu Cheng, MD, PhD,^1^ Ya-Ping Yang, MD, PhD,^1^ Fen Wang, MD,^2^ Ying-Chun Zhang, MD, PhD,^3^ Jing Chen, MD, PhD,^1^ Cheng-Jie Mao, MD, PhD^1^ and Chun-Feng Liu, MD, PhD,^1,2*^

^1^Department of Neurology, the Second Affiliated Hospital of Soochow University, Suzhou, Jiangsu, China

^2^Institute of Neuroscience, Soochow University, Suzhou, Jiangsu, China

^3^Department of Ultrasound, the Second Affiliated Hospital of Soochow University, Suzhou, Jiangsu, China

^†^Kai Li and Yi-Lun Ge contributed equally to this work.

***Correspondence Author:**

Dr. Chun-Feng Liu, MD, PhD

Department of Neurology, the Second Affiliated Hospital of Soochow University, No.1055 Sanxiang Road, Suzhou, Jiangsu, 215004, P. R. China

Tel.: (86)512-6778-3307

E-mail: liuchunfeng@suda.edu.cn

**Supplemental Table 2.** Genotypes of 34 common SNPs among 221 individuals with PD.

|  | Genotype | Total (n=221) | SN+ (n=122) | SN- (n=99) | OR | 95% CI | *P*^†^ |
| --- | --- | --- | --- | --- | --- | --- | --- |
| Gender (Male, %) |  | 138 (62.4%) | 92 (75.4%) | 46 (46.5%) | 0.32 | 0.16-0.63 | 0.001 |
| Age (years) |  | 62.5 ± 8.6 | 63.4 ± 8.2 | 61.4 ± 9.0 | 1.02 | 0.98-1.06 | 0.260 |
| PD Duration (years) |  | 4.3 ± 3.6 | 4.3 ± 3.8 | 4.2 ± 3.2 | 1.01 | 0.91-1.13 | 0.833 |
| H-Y |  | 2.1 ± 0.7 | 2.1 ± 0.7 | 2.1 ± 0.8 | 0.78 | 0.42-1.46 | 0.432 |
| UPDRS-III |  | 25.0 ± 11.9 | 25.6 ± 12.7 | 24.4 ± 10.9 | 1.01 | 0.97-1.05 | 0.552 |
| rs8177186 | GG/GT/TT | 136/69/16 | 68/45/9 | 68/24/7 | 1.89 | 0.83-4.30 | 0.129 |
| rs1130459 | GG/GA/AA | 129/71/21 | 70/44/8 | 59/27/13 | 1.02 | 0.36-2.88 | 0.964 |
| rs8177221 | GG/GA/AA | 152/58/11 | 83/36/3 | 69/22/8 | 0.77 | 0.19-3.04 | 0.707 |
| rs12769 | GG/GA/AA | 75/102/44 | 39/62/21 | 36/40/23 | 0.55 | 0.18-1.71 | 0.305 |
| rs4241357 | TT/TG/GG | 125/76/20 | 66/47/9 | 59/29/11 | 1.32 | 0.42-4.19 | 0.636 |
| rs1799852 | CC/CT/TT | 125/81/15 | 67/49/6 | 58/32/9 | 0.68 | 0.19-2.44 | 0.556 |
| rs3811658 | CC/CT/TT | 81/101/39 | 40/63/19 | 41/38/20 | 1.62 | 0.39-6.67 | 0.505 |
| rs1880669 | TT/TC/CC | 70/105/46 | 36/61/25 | 34/44/21 | 1.23 | 0.34-4.39 | 0.749 |
| rs1049296 | CC/CT/TT | 120/80/21 | 68/45/9 | 52/35/12 | 1.25 | 0.38-4.10 | 0.712 |
| rs224567 | AA/AG/GG | 78/116/27 | 42/65/15 | 36/51/12 | 1.18 | 0.56-2.52 | 0.663 |
| rs224454 | CC/CT/TT | 60/107/54 | 35/60/27 | 25/47/27 | 0.95 | 0.49-1.83 | 0.872 |
| rs2230267 | TT/TC/CC | 67/108/46 | 39/60/23 | 28/48/23 | 0.79 | 0.49-1.27 | 0.327 |
| rs2076114 | CC/CT/TT | 150/66/5 | 82/35/5 | 68/31/0 | 0.81 | 0.23-2.86 | 0.744 |
| rs3827354 | TT/TC/CC | 134/63/24 | 78/33/11 | 56/30/13 | 0.97 | 0.51-1.83 | 0.914 |
| rs3788533 | CC/CG/GG | 115/95/11 | 65/52/5 | 50/43/6 | 0.53 | 0.06-4.61 | 0.561 |
| rs4375 | TT/TC/CC | 111/97/13 | 62/53/7 | 49/44/6 | 1.78 | 0.20-16.01 | 0.607 |
| rs1005529 | TT/TC/CC | 154/56/11 | 91/26/5 | 63/30/6 | 0.60 | 0.26-1.37 | 0.224 |
| rs2284060 | TT/TC/CC | 116/89/16 | 64/47/11 | 52/42/5 | 2.01 | 0.76-5.29 | 0.159 |
| rs731821 | CC/CA/AA | 122/87/12 | 75/44/3 | 47/43/9 | 0.45 | 0.24-0.86 | 0.016 |
| rs3737084 | CC/CG/GG | 132/74/15 | 62/49/11 | 70/25/4 | 2.07 | 1.17-3.67 | 0.013 |
| rs73610117 | CC/CT/TT | 149/61/11 | 81/35/6 | 68/26/5 | 1.11 | 0.61-1.99 | 0.739 |
| rs10424582 | GG/GA/AA | 93/106/22 | 52/59/11 | 41/47/11 | 0.90 | 0.49-1.65 | 0.726 |
| rs1864141 | GG/GA/AA | 120/72/29 | 65/42/15 | 55/30/14 | 0.96 | 0.55-1.65 | 0.873 |
| rs598126 | AA/AG/GG | 85/109/27 | 46/63/13 | 39/46/14 | 0.81 | 0.49-1.33 | 0.405 |
| rs41294530 | AA/AG/GG | 124/84/13 | 69/46/7 | 55/38/6 | 1.08 | 0.51-2.29 | 0.838 |
| rs7516939 | CC/CT/TT | 94/95/32 | 49/57/16 | 45/38/16 | 0.96 | 0.49-1.89 | 0.911 |
| rs1288362 | TT/TC/CC | 141/62/18 | 70/40/12 | 71/22/6 | 1.32 | 0.77-2.27 | 0.320 |
| rs2301865 | AA/AG/GG | 92/104/25 | 55/53/14 | 37/51/11 | 0.83 | 0.49-1.42 | 0.502 |
| rs2074629 | CC/CT/TT | 125/86/10 | 61/54/7 | 64/32/3 | 1.51 | 0.81-2.82 | 0.192 |
| rs16861582 | AA/AG/GG | 91/98/32 | 55/50/17 | 36/48/15 | 1.14 | 0.65-2.00 | 0.645 |
| rs16861636 | AA/AG/GG | 105/90/26 | 63/44/15 | 42/46/11 | 1.55 | 0.55-4.40 | 0.409 |
| rs3736282 | CC/CT/TT | 119/86/16 | 71/44/7 | 48/42/9 | 0.45 | 0.13-1.48 | 0.187 |
| rs192861143 | CC/CT/TT | 165/44/12 | 94/24/4 | 71/20/8 | 1.18 | 0.46-3.08 | 0.729 |
| rs3731981 | CC/CT/TT | 137/69/15 | 80/37/5 | 57/32/10 | 0.56 | 0.23-1.35 | 0.197 |

PD: Parkinson’s disease; SN+: Substantia nigra hyperechogenicity; SN-: Substantia nigra hypoechogenicity; OR: odds ratio; H-Y: Hoehn and Yahr stage ("off" state); UPDRS-III: Unified Parkinson Disease Rating Scale Part III ("on" state).

^†^*P*-values estimated from binary logistic regression models adjusted for age, gender, disease severity, and disease duration.
