# Supplementary material for: Substantia nigra echogenicity is associated with serum ferritin, gender and iron-related genes in Parkinson’s disease
Source: Sci Rep. 2020 May 26;10:8660. doi: 10.1038/s41598-020-65537-5 (PMC7250839; doi:10.1038/s41598-020-65537-5)
Supplement: Supplementary file 3 — Supplementary Table 3. [file 41598_2020_65537_MOESM3_ESM.docx]

Substantia nigra echogenicity is associated with serum ferritin, gender and iron-related genes in Parkinson's disease

Kai Li, MD, PhD,^1†^ Yi-Lun Ge, MD,^1†^ Chen-Chen Gu, MD,^1^ Jin-Ru Zhang, MD, PhD,^1^ Hong Jin, MD,^1^ Jiao Li, MD, PhD,^1^ Xiao-Yu Cheng, MD, PhD,^1^ Ya-Ping Yang, MD, PhD,^1^ Fen Wang, MD,^2^ Ying-Chun Zhang, MD, PhD,^3^ Jing Chen, MD, PhD,^1^ Cheng-Jie Mao, MD, PhD^1^ and Chun-Feng Liu, MD, PhD,^1,2*^

^1^Department of Neurology, the Second Affiliated Hospital of Soochow University, Suzhou, Jiangsu, China

^2^Institute of Neuroscience, Soochow University, Suzhou, Jiangsu, China

^3^Department of Ultrasound, the Second Affiliated Hospital of Soochow University, Suzhou, Jiangsu, China

^†^Kai Li and Yi-Lun Ge contributed equally to this work.

***Correspondence Author:**

Dr. Chun-Feng Liu, MD, PhD

Department of Neurology, the Second Affiliated Hospital of Soochow University, No.1055 Sanxiang Road, Suzhou, Jiangsu, 215004, P. R. China

Tel.: (86)512-6778-3307

E-mail: liuchunfeng@suda.edu.cn

**Supplemental Table 3.** Alleles of 34 common SNPs among 221 individuals with PD.

|  | Allele | Total (n=221) | SN+ (n=122) | SN- (n=99) | OR | 95% CI | *P*^†^ |
| --- | --- | --- | --- | --- | --- | --- | --- |
| Gender (Male, %) |  | 276 (62.4%) | 184 (75.4%) | 92 (46.5%) | 0.31 | 0.20-0.49 | <0.001 |
| Age (years) |  | 62.5 ± 8.6 | 63.4 ± 8.2 | 61.4 ± 9.0 | 1.02 | 0.99-1.05 | 0.139 |
| PD Duration (years) |  | 4.3 ± 3.5 | 4.3 ± 3.8 | 4.2 ± 3.2 | 1.01 | 0.94-1.09 | 0.791 |
| H-Y |  | 2.1 ± 0.7 | 2.1 ± 0.7 | 2.1 ± 0.7 | 0.79 | 0.53-1.19 | 0.264 |
| UPDRS-III |  | 25.0 ± 11.9 | 25.6 ± 12.7 | 24.4 ± 10.9 | 1.01 | 0.99-1.04 | 0.293 |
| rs8177186 | G/T | 341/101 | 181/63 | 160/38 | 1.98 | 0.88-4.47 | 0.099 |
| rs1130459 | G/A | 329/113 | 184/60 | 145/53 | 0.95 | 0.33-2.72 | 0.928 |
| rs8177221 | G/A | 362/80 | 202/42 | 160/38 | 0.75 | 0.22-2.53 | 0.643 |
| rs12769 | G/A | 252/190 | 140/104 | 112/86 | 0.48 | 0.17-1.34 | 0.162 |
| rs4241357 | T/G | 326/116 | 179/65 | 147/51 | 1.68 | 0.62-4.50 | 0.305 |
| rs1799852 | C/T | 331/111 | 183/61 | 148/50 | 0.63 | 0.29-1.37 | 0.243 |
| rs3811658 | C/T | 263/179 | 143/101 | 120/78 | 1.75 | 0.53-5.81 | 0.359 |
| rs1880669 | T/C | 245/197 | 133/111 | 112/86 | 1.22 | 0.44-3.34 | 0.705 |
| rs1049296 | C/T | 320/122 | 181/63 | 139/59 | 1.06 | 0.50-2.26 | 0.882 |
| rs224567 | A/G | 272/170 | 149/95 | 123/75 | 1.23 | 0.54-2.77 | 0.621 |
| rs224454 | C/T | 227/215 | 130/114 | 97/101 | 0.85 | 0.40-1.79 | 0.664 |
| rs2230267 | T/C | 242/200 | 138/106 | 104/94 | 0.74 | 0.45-1.23 | 0.242 |
| rs2076114 | C/T | 366/76 | 199/45 | 167/31 | 0.92 | 0.35-2.41 | 0.868 |
| rs3827354 | T/C | 331/111 | 189/55 | 142/56 | 0.97 | 0.50-1.90 | 0.936 |
| rs3788533 | C/G | 325/117 | 182/62 | 143/55 | 0.39 | 0.04-4.08 | 0.432 |
| rs4375 | T/C | 319/123 | 177/67 | 142/56 | 2.43 | 0.24-24.63 | 0.453 |
| rs1005529 | T/C | 364/78 | 208/36 | 156/42 | 0.56 | 0.24-1.30 | 0.176 |
| rs2284060 | T/C | 321/121 | 175/69 | 146/52 | 2.19 | 0.94-5.12 | 0.071 |
| rs731821 | C/A | 331/111 | 194/50 | 137/61 | 0.44 | 0.24-0.83 | 0.011 |
| rs3737084 | C/G | 338/104 | 173/71 | 165/33 | 2.51 | 1.39-4.53 | 0.002 |
| rs73610117 | C/T | 359/83 | 197/47 | 162/36 | 1.11 | 0.59-2.06 | 0.753 |
| rs10424582 | G/A | 292/150 | 163/81 | 129/69 | 1.05 | 0.61-1.82 | 0.858 |
| rs1864141 | G/A | 312/130 | 172/72 | 140/58 | 1.02 | 0.61-1.72 | 0.940 |
| rs598126 | A/G | 279/163 | 155/89 | 124/74 | 0.81 | 0.48-1.35 | 0.411 |
| rs41294530 | A/G | 332/110 | 184/60 | 148/50 | 1.09 | 0.52-2.25 | 0.822 |
| rs7516939 | C/T | 283/159 | 155/89 | 128/70 | 0.94 | 0.50-1.79 | 0.857 |
| rs1288362 | T/C | 344/98 | 180/64 | 164/34 | 1.54 | 0.87-2.74 | 0.140 |
| rs2301865 | A/G | 288/154 | 163/81 | 125/73 | 0.81 | 0.48-1.38 | 0.439 |
| rs2074629 | C/T | 336/106 | 176/68 | 160/38 | 1.64 | 0.90-2.98 | 0.106 |
| rs16861582 | A/G | 280/162 | 160/84 | 120/78 | 0.98 | 0.55-1.74 | 0.941 |
| rs16861636 | A/G | 300/142 | 170/74 | 130/68 | 1.22 | 0.43-3.47 | 0.706 |
| rs3736282 | C/T | 324/118 | 186/58 | 138/60 | 0.60 | 0.19-1.89 | 0.379 |
| rs192861143 | C/T | 374/68 | 212/32 | 162/36 | 1.08 | 0.40-2.90 | 0.885 |
| rs3731981 | C/T | 343/99 | 197/47 | 146/52 | 0.55 | 0.23-1.32 | 0.181 |

PD: Parkinson’s disease; SN+: Substantia nigra hyperechogenicity; SN-: Substantia nigra hypoechogenicity; OR: odds ratio; H-Y: Hoehn and Yahr stage ("off" state); UPDRS-III: Unified Parkinson Disease Rating Scale Part III ("on" state).

^†^*P*-values estimated from binary logistic regression models adjusted for age, gender, disease severity, and disease duration.
